# Pharmacological Effects of Active Compounds on Neurodegenerative Disease with Gastrodia and Uncaria Decoction, a Commonly Used Poststroke Decoction

**DOI:** 10.1155/2013/896873

**Published:** 2013-11-14

**Authors:** Stanley C. C. Chik, Terry C. T. Or, D. Luo, Cindy L. H. Yang, Allan S. Y. Lau

**Affiliations:** Molecular Chinese Medicine Laboratory, Li Ka Shing Faculty of Medicine, The University of Hong Kong, Hong Kong Special Administrative Region, Hong Kong

## Abstract

Neurodegenerative diseases refer to the selective loss of neuronal systems in patients. The diseases cause high morbidity and mortality to approximately 22 million people worldwide and the number is expected to be tripled by 2050. Up to now, there is no effective prevention and treatment for the neurodegenerative diseases. Although some of the clinical therapies target at slowing down the progression of symptoms of the diseases, the general effectiveness of the drugs has been far from satisfactory. Traditional Chinese medicine becomes popular alternative remedies as it has been practiced clinically for more than thousands of years in China. As neurodegenerative diseases are mediated through different pathways, herbal decoction with multiple herbs is used as an effective therapeutic approach to work on multiple targets. Gastrodia and Uncaria Decoction, a popular TCM decoction, has been used to treat stroke in China. The decoction contains compounds including alkaloids, flavonoids, iridoids, carotenoids, and natural phenols, which have been found to possess anti-inflammatory, antioxidative, and antiapoptotic effects. In this review, we will summarize the recent publications of the pharmacological effects of these five groups of compounds. Understanding the mechanisms of action of these compounds may provide new treatment opportunities for the patients with neurodegenerative diseases.

## 1. Introduction

### 1.1. Neurodegenerative Diseases

Neurodegenerative diseases are associated with high morbidity, mortality, and economic burden to the community worldwide. It is estimated to have 22 million people worldwide suffering from these diseases and this number is expected to be triple in western countries by 2050 due to the increase in average life expectancy [1]. The diseases can be categorized as acute (including stroke, brain trauma, and spinal cord injury) and chronic (including amyotrophic lateral sclerosis (ALS), Huntington's disease, Alzheimer's disease (AD), and Parkinson's disease (PD)) diseases, which are characterized by selective loss of neuronal systems. Increasing evidence shows that neurodegenerative diseases are mediated by neuroinflammation, oxidative stress, and apoptotic cell death [2, 3].

#### 1.1.1. Role of Neuroinflammation in Neurodegenerative Diseases

Neuroinflammation is the reaction of the endogenous central nervous system (CNS) in response to various pathologic events. Upon stimulation, microglia, the resident tissue macrophages, are firstly activated to represent the first line of defense [4] by releasing proinflammatory mediators including cytokines, chemokines, proteases, and reactive oxygen species (ROS)/reactive nitrogen species [5–7]. Indeed, microglial activation is beneficial to the CNS as it controls infection, minimizes further injury, and promotes repair. Under other circumstances, however, irresolvable stimuli can cause highly detrimental effects to neurons by the excess productions of proinflammatory mediators including interleukin-1 (IL-1), IL-6, nitric oxide, monocyte chemotactic protein-1 (MCP-1), prostaglandins, and tumor necrosis factor-*α* (TNF-*α*) by microglial cells [8, 9]. The stimuli that cause microglial overactivation can be diverse, ranging from endotoxin (e.g., lipopolysaccharide (LPS)) and neurotoxin (e.g., amyloid-*β* (A**β**)) to neuronal death. A**β**, implicated in pathogenesis of AD, activates microglia to release neurotoxic factors such as nitric oxide [10] and TNF [11].

Interleukin-1 (IL-1), existing in two distinct isoforms, IL-1*α* and IL-1*β*, is one of the most widely studied proinflammatory cytokines in the brain. It is partly due to the elevated IL-1 expression detected in patients with acute or chronic neurodegenerative diseases [12]. Upon CNS damage/infection, microglial cells produce the highest levels of IL-1 [13]. In turn, IL-1 activates microglial and endothelial cells to produce various kinds of mediators including proinflammatory cytokines (IL-6 and TNF-*α*), chemokines (MCP-1), adhesion molecules, prostaglandins, ROS, nitric oxide, and matrix metalloproteases, which are considered to be toxic to neurons and glial cells [14]. 

Tumor necrosis factor-*α* (TNF-*α*) is another major mediator known to be implicated in several neurodegenerative diseases including AD, ALS, PD, and stroke [15]. In the CNS, elevated TNF-*α* is synthesized by astrocytes, neurons, and microglia upon infections/injuries [16]. TNF-*α* is found to mediate its effect via its receptor TNF-R1 which augments neuronal death through induction of caspases signalings [17]. Moreover, TNF-*α* can induce the productions of IL-1 and IL-6, which can amplify the neuroinflammation process.

Endotoxin and neurotoxin-induced proinflammatory cytokines productions have been regulated by three well-known mitogen-activated protein kinases (MAPKs) including extracellular signal-regulated kinase (ERK), p38, and c-Jun N-terminal kinase/stress-activated protein kinase (JNK) [18, 19]. MAPKs transduce the extracellular stimuli through a cascade of protein phosphorylations, which leads to the activation of transcription factor nuclear factor (NF)-*κ*B. The activated NF-*κ*B is then translocated and bound to *κ*B binding sites in the nucleus, leading to the initiation of transcription of proinflammatory mediators.

#### 1.1.2. Role of Oxidative Stress in Neurodegenerative Diseases

Oxidative stress refers to the imbalance between the production of ROS and the ability of the cells to eliminate them [20]. Numerous studies have provided evidence that ROS are directly involved in oxidative damage of cellular macromolecules, resulting in neuronal cell death [21]. ROS including hydrogen peroxide, nitric oxide, superoxide anion, and the highly reactive hydroxyl radicals are produced by microglia in response to the stimulation by endotoxin (e.g., LPS) [22], neurotoxin (e.g., A**β** and 6-hydroxydopamine (6-OHDA)) [23, 24], and environmental toxins (e.g., Rotenone) [25]. From which, nitric oxide can react with superoxide anion to form extremely reactive peroxynitrite, which can indiscriminately damage neurons by promoting membrane lipid peroxidation and by the formation of nitrotyrosine [26]. Furthermore, it stimulates the release of apoptosis-inducing factor (AIF) from the mitochondria, which subsequently triggers DNA fragmentation processes [27]. Enzymatic antioxidants including superoxide dismutase (SOD) and catalase (CAT) have been found to catalyze the dismutation of the superoxide anion and breakdown of hydrogen peroxide, respectively [28].

#### 1.1.3. Cell Death Mechanisms

Increasing lines of evidence indicate that neuronal cell death that occurred in various neurodegenerative disorders is mediated by necrosis and apoptosis [29]. Necrosis is a form of traumatic cell death by cytoplasmic swelling, nuclear dissolution, and lysis, while apoptosis is an orderly and compartmental dismantling of cells. In acute neurodegenerative diseases, like ischemic stroke, cells undergo necrosis in the core region of the ischemic infarct when their intracellular supply of metabolic substrates (e.g., glucose and oxygen) is depleted. The process triggers the generation of free radicals, glutamate, cytotoxic cytokines, and massive calcium influx through *N*-methyl-d-aspartate (NMDA) receptors and voltage-dependent calcium channels [30]. The calcium influx and ROS generation trigger the pathogenesis of apoptosis [31]. It is executed by caspases and regulated by the Bcl-2 protein family [32]. Caspases are considered as the major executioners of the apoptotic pathway [32]. Caspases-3, -6, and -7 are short prodomain caspases predominantly activated through the action by other proteases. Activated caspase-3 can cleave the DNA repair enzyme poly(ADP-ribose) polymerase (PARP), which can cause abrogation of DNA repair and depletion of NAD^+^ and ATP, resulting in cell death [33]. Apoptosis is also regulated by the Bcl-2 family of proteins [34], from which gene expressions are activated including pro- (Bad and Bax) and anti- (Bcl-2 and Bcl-xL) apoptotic proteins. In response to apoptotic stimuli, Bad and Bax translocate to mitochondria to initiate the release of cytochrome c, which is able to trigger the apoptotic signaling cascade by activating the caspase-9 and caspase-3, resulting in nuclear fragmentation and cell death [32]. In contrast, antiapoptotic proteins including Bcl-2 and Bcl-xL function as repressors of cell death in the CNS due to their abilities to block the release of cytochrome c suppress the accumulation of ROS [35]. 

### 1.2. TCM as an Alternative Medicine for Neurodegenerative Disease

Nowadays, there are no therapeutic drugs that can cure AD and PD due to the incomplete understanding of their pathogenic mechanisms. Current clinical practices to treat AD include the usage of cholinesterase inhibitors and glutamate modulators [36]. In addition, other approaches including anti-inflammatory, antioxidative, and anti-A**β**-peptides agents have been used to mitigate the symptoms of AD [37]. Recent therapies for PD are based mainly on three strategies: (1) the usage of L-DOPA for compensating the progressive degeneration of dopaminergic neurons in the substantia nigra pars compacta [38]; (2) the identification of non-dopaminergic drugs for alleviating nonmotor symptoms; and (3) the development of neuroprotective and disease-modifying compounds [39, 40]. Most of these clinical therapies on slowing down the progression of symptoms of the neurodegenerative diseases are still in the preclinical phase and only a few of them are in the clinical phase. However, the efficacies of the treatments are not considered as satisfactory [1]. For the treatment of acute ischemic stroke, thrombolytic agent such as recombinant tissue-plasminogen activator is available for the patients. However, the treatments are limited by a short therapeutic window of time and side effects [41]. At present, there is no effective clinical treatment that can enhance the recovery or prevent the recurrence of stroke. It is, therefore, critical to identify the pharmacological agents that can alleviate the neurodegenerative syndromes with fewer side effects compared with the current therapies.

Traditional Chinese medicine (TCM) has been practiced in health care systems for thousands of years in China. A variety of studies have demonstrated that the herbal formulations, herbal extracts, and active compounds derived from Chinese herbs are effective on the *in vitro* and *in vivo* neurodegenerative models [42, 43]. Since the pathogenesis of neurodegenerative diseases can not be single factor-derived, a combination of different herbs as herbal decoction could be an effective therapeutic approach. Herbal decoction consists of multiple crude herbs may work on multiple targets to enhance the therapeutic effects synergistically [40].

### 1.3. Pharmacological Effects of Gastrodia and Uncaria Decoction (GUD) on Neurodegenerative Disease

Gastrodia and Uncaria Decoction (GUD) is often used in TCM prescriptions for stroke treatment in China. It was first described in TCM book for clinical diagnosis and therapy, titled “New Significance of Patterns and Treatment in Miscellaneous Diseases.” There are 11 herbs in this decoction, including *Rhizoma Gastrodiae *(Tian Ma), *Ramulus cum Uncis Uncariae *(Gou Teng), *Concha Haliotidis *(Shi Jue Ming), *Fructus Gardeniae *(Zhi Zi), *Radix Scutellariae *(Huang Qin), *Eucommia ulmoides Oliver *(Du Zhong), *Radix Cyathulae Officinalis *(Niu Xi), *Ramulus Loranthi *(Sang Ji Sheng), *Poria cum Radix Pini *(Fu Sheng), *Caulis Polygoni Multiflori *(Ye Jiao Teng), and *Herba Leonuri *(Yi Mu Cao). For the stroke treatment, the pharmacological effects of the decoction are (1) to subdue the liver and extinguish internal wind (Tian Ma, Gou Teng, and Shi Jue Ming); (2) to entice the blood downward (Niu Xi); (3) to purge liver fire (Huang Qin and Zhi Zi); (4) to supplement liver and kidney (Sang Ji Sheng and Du Zhong); (5) to vitalize the blood and enhance water metabolism (Yi Mu Cao); and (6) to pacify the restless heart (Ye Jiao Teng and Fu Sheng) [44].

In addition, a study revealed that GUD significantly prevented hypertension in spontaneously hypertensive rats (SHR) model and altered the development of hypertension [45]. Another study reported that the serum of GUD-treated rat inhibited the proliferation of vascular smooth muscle cells (VSMCs) by suppressing the expressions of proliferating cell nuclear antigen (PCNA) and c-myc, increasing nitric oxide and decreasing endothelin-1 (ET-1) levels [46].

**Figure 1:**
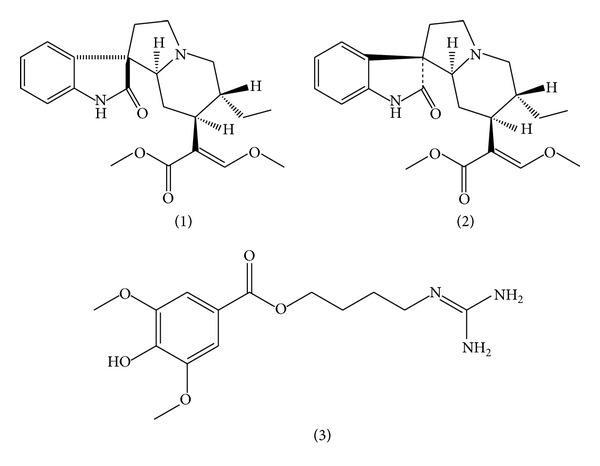
Chemical structure of alkaloids: (1) rhynchophylline; (2) isorhynchophylline; (3) leonurine.

**Figure 2:**
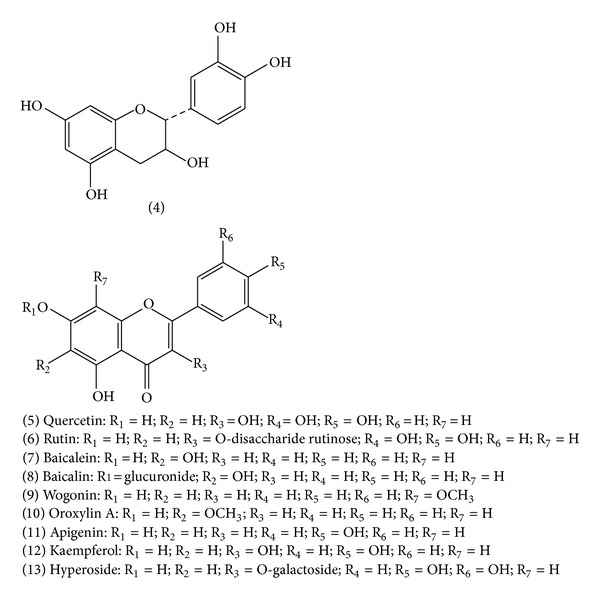
Chemical structure of flavonoids: (4) catechin; (5) quercetin; (6) rutin; (7) baicalein; (8) baicalin; (9) wogonin; (10) oroxylin A; (11) apigenin; (12) kaempferol; (13) hyperoside.

**Figure 3:**
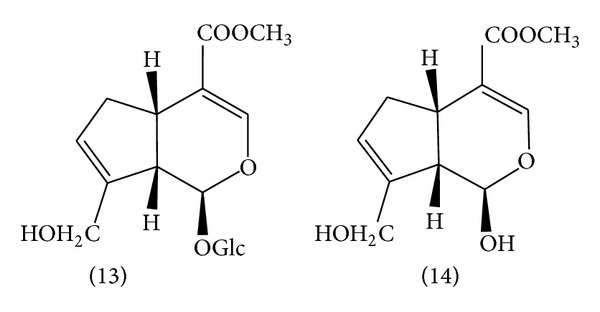
Chemical structure of iridoids: (13) geniposide and (14) genipin.

**Figure 4:**
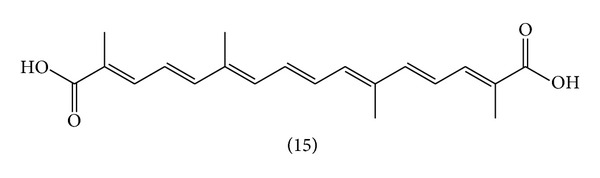
Chemical structure of carotenoid: (15) crocetin.

**Figure 5:**
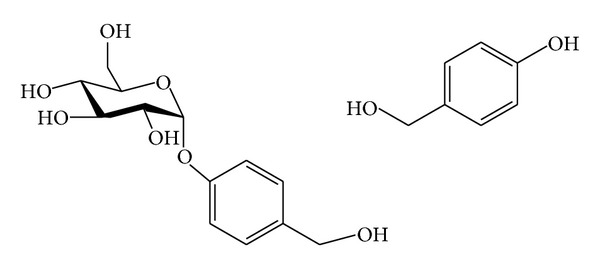
Chemical structure of phenolic compounds: (16) gastrodin and (17) *p*-Hydroxybenzyl alcohol.

**Figure 6:**
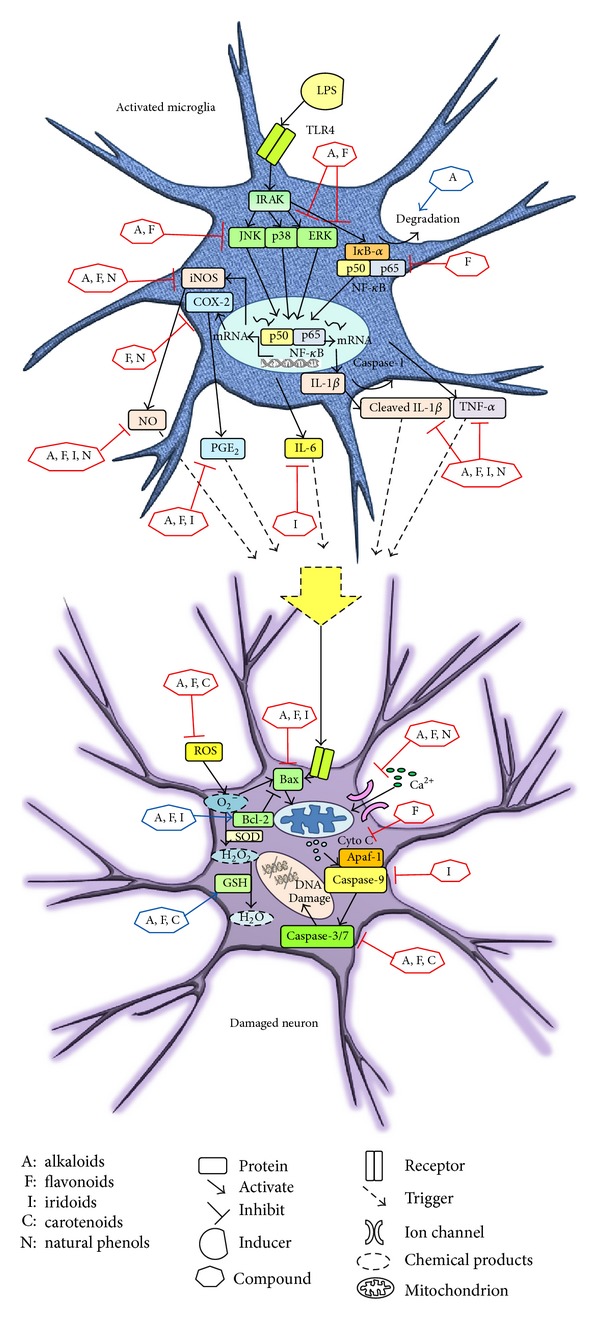
Summary of the effects of different groups of compounds from GUD on the signaling pathways involved in inflammatory responses in microglia and apoptosis in neuronal cells in neurodegenerative diseases.

Neurodegenerative disorders and stroke share many pathogenic mechanisms such as inflammation, microglial activation, oxidative stress, impaired neurotransmission, mitochondrial dysfunction, and apoptosis [47, 48]. Neuroprotective agents that interfere with the biochemical cascades would be the potential therapeutic candidates. Thus, the combination of different active compounds from individual herb aiming at specific pathological factors would be the systematic approach for the treatment of neurodegenerative diseases. In this review, the anti-inflammatory, antioxidative, and antiapoptotic effects of some of the active pharmacological compounds (Figures 1, 2, 3, 4, and 5) identified from individual herb of GUD will be summarized (Table 1 and Figure 6).

## 2. Alkaloids

Alkaloids, a group of naturally occurring compounds originated from plant and microbes, contain at least one nitrogen atom in the structure. The functions of alkaloids are well known to protect the plants from predators and regulate plant growth. Its pharmacological activities have been documented to be anticancer [49], anti-inflammatory [50], and cardiovascular effects [51].

### 2.1. Rhynchophylline and Isorhynchophylline

Rhynchophylline and isorhynchophylline are the two most dominant alkaloid constituents in *Ramulus cum Uncis Uncariae* [52, 53]. Their pharmacological activities were extensively studied which have been shown to exert anti-inflammatory [54–56], antioxidative [57, 58], and neuroprotective [57, 59–64] effects in CNS. Anti-inflammatory effects of rhynchophylline and isorhynchophylline were found in primary cultured rat cortical microglia. One of the investigations showed that both rhynchophylline and isorhynchophylline reduced the production of nitric oxide in LPS-induced rat cortical microglia [54]. More recently, another study investigated the action of rhynchophylline on the productions of proinflammatory mediators and its potential mechanisms in LPS-activated microglia. The study showed that rhynchophylline reduced the productions of nitric oxide, prostaglandin E2 (PGE_2_), MCP-1, TNF-*α*, and IL-1*β* [56]. Besides, mRNA levels of inducible nitric oxide synthase (iNOS) and cyclo-oxygenase-2 (COX-2) were also suppressed by rhynchophylline [56]. The molecular mechanisms for the inhibitions were due to the blocking of the NF-*κ*B activation and the suppression of ERK and p38 phosphorylations in activated microglia [56]. Apart from rat primary microglia, rhynchophylline and isorhynchophylline effectively suppressed the release of nitric oxide, TNF-*α*, and IL-1*β* via suppressions of iNOS protein level, ERK and p38 phosphorylations, and degradation of I*κ*B*α* in LPS-activated murine N9 microglial cell line, with isorhynchophylline showing more potent inhibition [55].

Isorhynchophylline exerted protective effect upon oxidative stress. In a recent study, isorhynchophylline was found to possess neuroprotective effects against A**β**-induced neurotoxicity in PC12 cells [58]. The protections were due to the reduction of intracellular ROS and malondialdehyde (MDA) levels, induction of glutathione (GSH) level, and stabilization of mitochondrial membrane potential [58]. In addition, isorhynchophylline suppressed the mitochondrial pathway of cellular apoptosis by reducing the formation of DNA fragmentation and the activity of caspase-3 as well as moderating the ratio of Bcl-2/Bax expression [58].

Rhynchophylline was found to have neuroprotective effects upon the induction of neurotoxin. Dopamine-induced apoptosis of NT2 neurons has been suppressed by rhynchophylline [61]. In another study using rat primary cortical neurons, rhynchophylline protected against methamphetamine (MA)-induced neurotoxicity [63]. Excessive activation of NMDA subtype glutamate receptors by glutamate triggered neuronal cell damage in the brain [65]. Rhynchophylline and isorhynchophylline were shown to suppress glutamate-induced neuronal death in rat cerebellar granule cells by inhibition of Ca^2+^ influx [59] and they both acted as noncompetitive antagonists of NMDA receptor expressed in *Xenopus* oocytes [60]. Further investigation using deprivation of oxygen and glucose-induced neuronal damage in rat hippocampus showed that both rhynchophylline and isorhynchophylline suppressed the *in vitro* ischemia-induced neuronal damage [62]. The inhibitory effects might attribute to the interactions of rhynchophylline and isorhynchophylline with neurotransmitters receptors other than the NMDA subtype such as muscarinic M_1_ and 5-HT_2_ receptors [62].

Aberrant expressions and aggregation of *α*-synuclein (*α*-syn) in neurons are the pathogenic factors of PD [66]. A recent study suggested that isorhynchophylline induced autophagy in different neuronal cell lines including N2a, SH-SY5Y, and PC12 cells and also in primary cortical neurons [64]. The induction of autophagy was due to the promotion of clearance of wild-type, A53T and A30P *α*-syn monomers, *α*-syn oligomers, and *α*-syn/synphilin-1 aggresomes in neuronal cells via the autophagy-lysosome pathway [64]. Besides, isorhynchophylline reduced the wild-type and A53T *α*-syn protein expressions in differentiated human dopaminergic neurons [64].

The anti-inflammatory effect of rhynchophylline has been demonstrated in an *in vivo* model. In a kainic acid-induced seizures rat model, rhynchophylline has the antiepileptic effects associated with the reduction of the superoxide anions levels, JNK phosphorylation, and NF-*κ*B activation [57]. 

### 2.2. Leonurine

Leonurine, another alkaloid present in *Herba Leonuri*, has been demonstrated as an effective cardiovascular agent in preclinical studies. Its neuroprotective effect on neuronal cells has also been documented [67–69]. In an *in vitro* study, leonurine protected SH-SY5Y cells from cytotoxicity and apoptosis induced by 6-OHDA [67]. The underlying mechanisms were due to its anti-inflammatory and antiapoptotic effects by ameliorating intracellular ROS generation, downregulating of proapoptotic Bax, and upregulating of antiapoptotic Bcl-2 in both mRNA and protein levels [67]. The therapeutic potential of leonurine on brain injury has been demonstrated using transient rat middle cerebral artery occlusion (MCAO) models. With the pretreatment of leonurine, the infarct volume of the neurological impairment of rats was found to be reduced after MCAO [68]. Moreover, the mitochondrial ROS was reduced as a result of leonurine treatment, indicating that leonurine possessed antioxidant activity to exert its antiapoptotic effect by preserving mitochondrial function [68]. A similar study also demonstrated the antioxidant effect of leonurine, which reduced the infarct volume and improved the neurological deficit of rats after MCAO [69]. At the same time, it also increased the activities of antioxidant enzymes including superoxide dismutase and glutathione peroxidase [69]. 

## 3. Flavonoids

Flavonoids represent the largest group of plant-specific secondary metabolites for flower coloration. They chemically consist of 15 carbon atoms arranged in C6-C3-C6 skeleton. They possess a wide spectrum of biological activities including anti-inflammatory, anticancer, and antioxidative effects [70]. The antioxidative effect may result from direct scavenging of ROS, high propensity to electron transfer, and chelating ferrous l ions by flavonoids [71].

### 3.1. Quercetin and Catechin

Quercetin and catechin are widely studied for their potential pharmacological properties on neurodegenerative diseases based on their underlying mechanisms related to anti-inflammatory [72, 73], antioxidative [74–77], and neuroprotective [76–83] effects on both *in vitro* and *in vivo* neural damage models. 

Quercetin was reported to have anti-inflammatory effects on LPS-induced N9 microglial cells by suppressing IL-1*α* and TNF-*α* mRNA levels [72]. Moreover, quercetin reduced inflammation-mediated apoptotic neuronal cell death in the microglial-neuronal coculture system [72]. In addition, quercetin inhibited overproduction of nitric oxide and overexpression of iNOS in dopaminergic neurotoxin 6-OHDA-induced PC12 cells [73].

Antioxidative effects of flavonoids associated with neural diseases have been extensively studied. In the rat primary cultures, both quercetin and catechin protected the mesencephalic cultures from oxidative insult by N-methyl-4-phenyl-1,2,3,6-tetrahydropyridinium hydrochloride (MPP^+^) [75]. Catechin increased the cellular viability and [^3^H] DA uptake by reducing the injury produced by hydrogen peroxide (H_2_O_2_), 4-hydroxynonenal (4-HNE), rotenone, and 6-OHDA [75]. Most recently, a study using P19 neurons obtained by the differentiation from mouse teratocarcinoma P19 cells was investigated. The study showed that quercetin increased the neuronal viability by diminishing ROS generation and inhibiting nuclear condensation, caspases-3 and -7 activities, and poly(APD-ribose) polymerase upregulation under H_2_O_2_-induced oxidative stress [76].

Flavonoids were found to possess antioxidant activities [84] and exert protective effects on neuronal cells from oxidative stress-induced neurotoxicity [75, 85]. Catechin was proven to exert protective effects against glutamate-induced neuronal death in cultured rat cerebellar granule cells by inhibiting Ca^2+^ influx [78]. Besides, more investigations were focused on the neuroprotective effects of quercetin in neuronal cells. One of the studies using rat cortical neuronal cultures showed that quercetin significantly attenuated A*β*_(1–42)_-induced cytotoxicity, protein oxidation, lipid peroxidation, and apoptosis [79]. Apart from the primary cultures, oxidative stress was applied to neuronal cell lines to evaluate the effects of quercetin on the apoptotic cascades. Quercetin was found to suppress the apoptotic neuronal cell death by inhibiting the activation of caspase cascade through moderating the expression of proapoptotic gene (Bax) and antiapoptotic gene (Bcl-2) in MPP^+^- and H_2_O_2_-induced PC12 and SH-SY5Y cells, respectively [80, 81].

Quercetin and catechin have been investigated to demonstrate their neuroprotective properties in the *in vivo* model. Catechin was found to attenuate monoamine oxidase B (MAO-B) activity in rat brain which provided protection against oxidative neurodegeneration [74]. In a repeated cerebral ischemia rat model, quercetin improved spatial memory impairment and decreased neuronal cell death in the hippocampal CA1 area [82]. Moreover, liposomal quercetin reduced cerebral damage provoked by permanent MCAO (pMCAO) by protecting against ischemic lesions and increasing GSH levels in ipsilateral striatum and cortex in rats [83]. Besides, in the 6-OHDA-induced rat model of PD, quercetin defended against oxidative stress and reduced dopaminergic neuronal loss by increasing striatal dopamine and antioxidant enzyme levels together with decreasing protein carbonyl content in striatum [77]. Recently, zebra fish has been used as a PD disease model. Quercetin prevented 6-OHDA-stimulated dopaminergic neuron loss by downregulating the overexpressions of IL-1*β*, TNF-*α*, and COX-2 in zebra fish [73].

Despite the extensive studies of quercetin in various *in vitro* and *in vivo* neural disease models, the neuroprotective effect of quercetin remains controversial due to its inability to cross the blood-brain barrier under *in vivo* conditions [86, 87]. More mechanistic studies have to be investigated in order to confer the conclusions.

### 3.2. Rutin

Rutin, comprising quercetin and disaccharide rutinose, is a member of flavonoids also called vitamin P present in *Herba Leonuri*. It is regarded as an important nutritional supplement due to the pharmacological properties including anticarcinogenic, cardioprotective, antioxidant, and anti-inflammatory activities [88]. The anti-inflammatory activity of rutin has been well documented in several studies. *In vitro*, rutin inhibited LPS-induced nitric oxide production and iNOS gene expression in a concentration-dependent manner in RAW 264.7 cells [89]. It was found to dose dependently attenuate A**β**_42_-induced cytotoxicity in SH-SY5Y neuroblastoma cells by inhibiting the formation of ROS and nitric oxide through the reduction of iNOS activity [90], suggesting that it can be used to treat AD. Furthermore, rutin modulated the production of proinflammatory cytokines including TNF-*α* and IL-1*β* in A**β**_42_-induced BV-2 microglia [90].

The neuroprotective effect of rutin was investigated in 6-OHDA-induced PD rat model. Rutin showed anti-inflammatory effect by reducing the productions of TNF-*α*, IL-1*β*, and nitric oxide as well as the expression of iNOS in 6-OHDA treated rats [91]. Moreover, rutin suppressed trimethyltin (TMT)-induced microglial activation by reducing the mRNA levels of IL-1*β* and IL-6, which resulted in the reduction of inflammation or neuron loss in the hippocampus of rats [92]. Another study also demonstrated that rutin reduced cerebral ischemia-induced neuronal death in the hippocampal region CA1 of rats [93]. The results illustrate that rutin is a potent anti-inflammatory agent for treating neurodegenerative diseases. 

### 3.3. Baicalein

Baicalein, a flavonoid originally isolated from the root of *Scutellaria baicalensis Georgi*, has various biological activities including anti-inflammatory [94], antioxidant, antiviral [95], and antifibrotic effects [96]. *In vitro*, baicalein possessed anti-inflammatory effect by inhibiting hypoxia-inducible factor-1 alpha (HIF-1*α*) protein accumulation and HIF-1 transcriptional activation on hypoxia-induced BV2 microglia [97]. Moreover, it suppressed the expressions of iNOS, COX-2, and VEGF by inhibiting ROS and PI3-kinase/Akt pathway on hypoxia-induced BV2 microglia [97]. Using LPS as an inducer, baicalein was found to almost completely block the activation of microglia by attenuating the excessive productions of TNF-*α* and free radicals including nitric oxide and superoxide [94]. Baicalein was also found to inhibit LPS/IFN-*γ*-induced nitric oxide production and activation of iNOS gene expression in primary microglia and BV-2 cells, through the inactivation of NF-IL6 [98].

In terms of antioxidative effect, baicalein protected the neuronal SH-SY5Y cells from 6-OHDA-induced cell apoptosis through the suppressions of caspase-3, caspase-9, and phospho-JNK activation as well as ROS generation [99, 100]. Baicalein protected HT22 mouse hippocampal neuronal cells against thapsigargin (TG) and brefeldin A (BFA)-induced apoptosis through the inhibition of ROS accumulation [101]. Moreover, a study demonstrated that baicalein inhibited ROS-mediated cytotoxic effects through the modulation of ERKs activation and the induction of heme oxygenase-1 (HO-1) protein expression on H_2_O_2_-induced rat glioma C6 cells [102]. Another study found that baicalein suppressed neurotoxin rotenone-induced apoptosis and inhibited the accumulation of ROS on rotenone-induced toxicity in PC12 cells [103].

*In vivo*, baicalein has been demonstrated to improve functional recovery and reduce contusion volumes of rats after controlled cortical impact injury [104]. Moreover, it also significantly reduced the mRNA and protein levels of proinflammatory cytokines including TNF-*α*, IL-1*β*, and IL-6 [104]. In general, the *in vitro* and *in vivo* data were suggestive of the potential role of baicalein in protecting the brain cells from apoptosis.

### 3.4. Baicalin

Baicalin, a flavonoid compound isolated from *Scutellaria baicalensis Georgi*, has been widely used in China to treat the inflammatory diseases and ischemic stroke for thousands of years. *In vitro*, RAW 264.7 cells induced by LPS have been used as a model to study the anti-inflammatory effect of baicalin. One of the studies demonstrated that baicalin inhibited nitric oxide production and iNOS gene expression [105]. Another study found that baicalin inhibited the productions of proinflammatory mediators including TNF-*α*, ET-1, and thromboxane A2 (TXA2) [106]. Moreover, baicalin possessed antioxidative effect by reducing ROS production, inhibiting 5-LOX translocation to the nuclear envelope, and inhibiting p38 phosphorylation in oxygen-glucose deprivation/H_2_O_2_-induced PC12 cells [107].

*In vivo*, baicalin was found to reduce neurological deficit scores and cerebral infarct volume of Sprague-Dawley rats after pMCAO [108]. The underlying mechanisms were due to the anti-inflammatory effect of baicalin that significantly reduced the expressions of iNOS and COX-2 mRNA levels in rat brain [108]. Moreover, it also possessed antioxidative effect to inhibit neuronal apoptosis and the expression of cleaved caspase-3 protein [108]. Tu et al. found that baicalin reduced the expression of TLR2/4 and NF-*κ*B and attenuated the serum content of proinflammatory cytokines including TNF-*α* and IL-1*β* [109]. Another study demonstrated that rats treated with baicalin show significant reduced neurological deficit scores and infarction volume [110]. Further investigations revealed that the neuroprotective effects of baicalin were due to the inhibition of NF-*κ*B p65 [110]. Similar study demonstrated that baicalin inhibited the transcription of NF-*κ*B and the generation of inflammatory mediator TNF-*α* in the injured spinal cord tissue of rats [111]. Moreover, baicalin reduced the expression of Bax and increased the expression of Bcl-2 [111].

### 3.5. Wogonin

Wogonin is one of the flavonoids from the root of* Scutellaria baicalensis *Georgi. The anti-inflammatory effect of wogonin has been recognized for a long time. A previous study demonstrated that the mechanisms of anti-inflammatory effect of wogonin involved the modulation of mediators and enzyme systems including cytokines, COX-2, and iNOS [112–116]. For instance, wogonin inhibited LPS-induced COX-2 expression and PGE_2_ production in RAW 264.7 cells [112]. In addition, wogonin decreased the productions of proinflammatory cytokines including TNF-*α* and IL-6 in LPS-induced microglia [116]. Furthermore, wogonin completely suppressed the activity of NF-*κ*B in MCP-1-stimulated microglia [117]. Wogonin was found to inhibit nitric oxide production through the suppression of iNOS on LPS-induced RAW 264.7 cells [118]. 

The neuroprotective effect of wogonin was demonstrated using animal models. Wogonin was found to reduce the total infarction volume and improve the behavioral deficits in rats after pMCAO [119]. Another study also demonstrated that wogonin exerted inhibitory activity on LPS-induced nitric oxide production through the suppression of iNOS expression in Balb/c mice [113].

### 3.6. Oroxylin A

Oroxylin A, being one of the principle components in* Scutellaria baicalensis *Georgi, possesses strong anti-inflammatory effect by inhibiting LPS/Bcl-induced iNOS and COX-2 gene expressions in RAW 264.7 cells [120]. The mechanism was due to the inhibition of the binding of transcription factor NF-*κ*B to the iNOS promoter [120]. Furthermore, oroxylin A inhibited 12-lipoxygenase in human platelets [121].

*In vivo*, pretreatment of oroxylin A ameliorated the memory impairment of A*β*_(25–35)_-induced mice, through the reduction of astrocyte and microglia activations and iNOS expression [122]. The result revealed that oroxylin A can be potentially used for treating PD.

### 3.7. Apigenin

The anti-inflammatory activity of apigenin, presents in *Herba Leonuri*, has been well studied. Apigenin inhibited the protein and mRNA expressions of COX-2 and iNOS in LPS-induced RAW 264.7 cells through the inhibition of I*κ*B kinase activity [123]. Apigenin also inhibited the productions of proinflammatory cytokines including TNF-*α*, IL-6, and IL-1*β* on LPS-stimulated human peripheral blood mononuclear cells [124]. It also reduced the mRNA levels of TNF-*α* and IL-1*β* in LPS-induced J774.2 macrophages [125]. Apigenin inhibited the production of nitric oxide and PGE_2_ by suppressing the expression of iNOS and COX-2 protein, respectively. Moreover, apigenin suppressed the phosphorylations of p38 and JNK but not the activity of ERK [126].

*In vivo*, apigenin significantly reduced the infarct volume of mice in MCAO model. Also, it inhibited microglial activation, resulting in the reduction of damage in various neurodegenerative diseases [126].

### 3.8. Kaempferol

Kaempferol can be found from *Herba Leonuri*, possessing anti-inflammatory activity as illustrated using LPS-induced J774.2 macrophages as the model. The compound was found to decrease the number of TNF-*α* mRNA copies and inhibit IL-1*β* gene expression in LPS-activated J774.2 macrophages [125]. Besides, it also effectively inhibited the productions of PGE_2_ and nitric oxide as well as downregulated microsomal PGE_2_ synthase-1 (mPGES-1) and iNOS expressions [127, 128]. The mechanisms were due to the inhibition of the activation of NF-*κ*B and signal transducer and activator of transcription 1 (STAT-1) [128]. In combination with another flavonoid chrysin, kaempferol was found to significantly synergize their inhibitory effect on nitric oxide, PGE_2_, and TNF-*α* secretions in LPS-induced RAW 264.7 macrophages [129]. The *in vivo* experiment demonstrated that kaempferol protected the rat brain from damage in the temporal-frontal areas of neocortex and striatum [130]. Kaempferol also protected the rat brain against nitrosative-oxidative stress after ischemia/reperfusion, as shown by nearly complete protection against the increase of protein nitrotyrosines, and also afford strong protection against the increase of apoptotic cell death and biochemical markers of apoptosis including caspase-9 activity and poly(ADP-ribose) polymerase degradation [130].

### 3.9. Hyperoside

Hyperoside (Hyp, quercetin-3-O-galactoside), a flavonoid compound, is usually found in *Herba Leonuri*. Hyperoside was shown to exert an anti-inflammatory action through suppressing the productions of TNF-*α*, IL-6, and nitric oxide in LPS-induced mouse peritoneal macrophages [131]. The antioxidative effect of hyperoside was demonstrated by investigating its effects on H_2_O_2_ and tert-butyl hydroperoxide-induced PC12 cytotoxicity. The result showed that hyperoside efficiently prevented PC12 cells from shrinking and turning against apoptosis as indicated by the decrease of extracellular lactate dehydrogenase levels [132]. The antiapoptotic effect of hyperoside was found to be partially dependent on suppressing the ROS production, inhibiting the caspase-3 activity [133]. It was also associated with increasing the expression of the antiapoptotic protein Bcl-2 and decreasing the expression of proapoptotic protein Bax in sodium azide-mediated PC12 cells [133]. *In vivo*, hyperoside reduced the infarct sizes and water content, and neurology score of the rat brain underwent focal cerebral ischemia reperfusion injury [134].

## 4. Iridoids

Iridoids, the secondary metabolites found in a wide variety of plants, are monoterpenes biosynthesized from isoprene. Chemically, they consist of a cyclopentane ring fused to a six-membered oxygen heterocycle. The iridoids produced by plants are primarily used to protect against herbivores or to prevent the plants against infection. This group of compound has attracted much interest for medicinal use because of their pharmaceutical activities including cardiovascular, anti-inflammatory, neuroprotective, antitumor, antiviral, and immunomodulatory activities [135].

### 4.1. Genipin and Geniposide

Iridoid compounds found in *Gardenia jasminoides* such as genipin and geniposide have been demonstrated to have diverse pharmacological activities including anti-inflammatory [136–138], antioxidative [139–142], neuroprotective [143–148], and neurotrophic [149–152] effects. Thus, the iridoid compounds have been considered to be the treatment of various neurological disorders. 

Genipin, the aglycone of geniposide, has been examined to evaluate its anti-inflammatory properties in the *in vitro* experiments. Genipin dose dependently inhibited the productions of NO and PGE_2_ as well as the expressions of iNOS, COX-2, IL-6, IL-1*β*, and TNF-*α* via downregulation of NF-*κ*B activation in LPS-stimulated RAW 264.7 macrophage [136]. Besides, microglial activations were reduced by genipin through the inhibitions of nitric oxide, TNF-*α*, IL-1*β*, PGE_2_, intracellular ROS productions, and NF-*κ*B activation in LPS-stimulated rat brain microglial cells [137]. Other than LPS, more pathophysiological stimuli A**β**in combination with IFN-*γ* were used to stimulate the microglial cells. Both pretreatment and posttreatment of genipin reduced nitric oxide release in A**β**and IFN-*γ* stimulated microglia [137].

Recently, genipin has been isolated from *Eucommia ulmoides *Oliver in our laboratory. The biological effect of genipin was studied and the results showed that genipin dose dependently inhibited the productions of nitric oxide and TNF-*α* as well as iNOS mRNA levels in LPS-stimulated BV-2 mouse microglial cells (data not shown). The inhibition was due to the suppressive effect of genipin on PI3K/Akt activation. Moreover, genipin protected Neuro-2a cells against neurotoxicity stimulated by the conditioned media transferred from LPS-challenged BV-2 cells.

Oxidative stress in brain is associated with the potential causative factor of age-related neurodegenerative disorders [153]. Compounds with capability to induce endogenous antioxidative proteins were considered as a therapeutic strategy for attenuating the oxidative damage and cell death in the brain. Geniposide was able to protect against 3-morpholinosydnonimine hydrochloride (SIN-1)-induced oxidative stress by enhancing HO-1 expression in both PC12 cells and primary hippocampal neurons [139, 142]. Geniposide also exerted protective effect against H_2_O_2_ by inducing Bcl-2 expression via MAPK and PI3K signaling pathways in PC12 cells [140, 141].

As the consequence of oxidative stress, induction of mitochondrial-mediated apoptosis is correlated with the development of neurodegenerative diseases. A number of studies focused on the regulation of mitochondrial pathways including the apoptosis-related protein expressions and the neurotoxin-induced neuronal cell death. Cobalt chloride (CoCl_2_)-induced cell death in PC12 cells, which mimic hypoxia-induced neuronal cytotoxicity, was protected by geniposide through decreasing the expression of Bax, P53, and caspase-9 while increasing the expression of Bcl-2 [147]. Besides, in the model of rat hippocampal slice culture, geniposide protected neuronal cell death from oxygen and glucose deprivation [148]. In addition, genipin protected neuronal cells against cytotoxicity induced by various neurotoxic agents including A*β*_(25–35)_ [145], 6-OHDA [144], A23187 (a calcium ionophore) [143], and tunicamycin [146].

Anti-inflammatory effects of genipin have been evaluated in the *in vivo* models. Neuroinflammation induced by systemic administration of LPS in mouse has been inhibited by genipin through the reduction of TNF-*α* and IL-6 productions in plasma [136]. Genipin could also provide neuroprotection by reducing the productions of various neurotoxic molecules from activated microglia [137]. Moreover, genipin effectively inhibited carrageenan-induced paw edema [136, 138], air pouch formation, and nitric oxide content in the exudates of mouse models [138].

## 5. Carotenoids

Carotenoids are tetraterpenoids naturally occurring in plants and other photosynthetic organisms. The structure of carotenoids is composed of a central carbon chain of alternating single and double bonds. The conjugated double bonds are well known to be responsible for the pigments of the carotenoids. They possessed antioxidant [154], cardiovascular [155], and anticancer [155] effects as well as reduced the risk of neurodegenerative diseases [156].

### 5.1. Crocetin

Crocetin is a natural carotenoid compound found in *Gardenia jasminoides*. Antioxidant effects of crocetin have been evaluated in both *in vitro* and *in vivo* models. In one model using stroke-prone spontaneously hypertensive rat (SHRSP), crocetin reduced oxidative stress in the isolated brain by acting as a scavenger of ROS [157]. In another study using SH-SY5Y cells, crocetin protected against cellular apoptosis by repressing ROS production and decreasing caspase-3 activation [158]. Neuromodulatory effect of crocetin has been investigated in the *in vivo* experiment. Report showed that pretreatment of crocetin enhanced the antioxidant enzyme activity, GSH, and dopamine levels whereas attenuated thiobarbituric acid reactive substance (TBARS) level in 6-OHDA-induced rats [159].

## 6. Phenolic Compounds

Phenolic compounds are believed to be one of the most widely occurring groups of phytochemicals throughout the plant kingdom. These compounds contain at least one aromatic ring with one or more hydroxyl groups attached. They contain hydrogen or electron-donating groups to interact with the radicals, resulting in antioxidant activities. 

### 6.1. Gastrodin

Gastrodin is the main component isolated from the rhizome of *Gastrodia elata*. This compound possessed strong anti-inflammatory and antioxidant effects [160–163]. *In vitro*, pretreatment of gastrodin has been demonstrated to reduce the neurotoxicity on hypoxia-induced rat cortical neurons [160]. It also protected against oxygen/glucose deprivation and glutamate-induced neuronal cell death in cultured rat hippocampal neurons by inhibiting Ca^2+^ and nitric oxide productions [161]. Gastrodin significantly reduced the protein and mRNA expression levels of iNOS, COX-2, TNF-*α*, and IL-1*β* in LPS-induced BV-2 cells [162]. Furthermore, levels of phosphorylated ERK1/2, JNK, and p38 MAPKs were significantly reduced by the pretreatment of gastrodin in LPS-stimulated microglial cells [162]. 

*In vivo*, gastrodin decreased the volume of cerebral infarction and ameliorated the cerebral injury in the rats of cerebral ischemia reperfusion [161, 163]. The studies revealed that gastrodin had a neuroprotective effect against neurodegenerative diseases.

### 6.2. *p*-Hydroxybenzyl Alcohol

p-Hydroxybenzyl alcohol is one of the major components in *Gastrodia elata *Blume (GE). It incorporated with copolyoxalate to exert excellent antioxidant activity by inhibiting nitric oxide production through the suppression of iNOS expression in LPS-activated RAW 264.7 cells [164]. Without copolyoxalate, p-Hydroxybenzyl alcohol inhibited nitric oxide production through the suppression of iNOS expression in LPS-activated BV-2 cells [165]. Moreover, p-Hydroxybenzyl alcohol also prevented PC12 cell death induced by H_2_O_2_ [166]. 

*In vivo*, p-Hydroxybenzyl alcohol provided neuroprotection by preventing brain damage through the increased expression of genes encoding antioxidant proteins including protein disulfide isomerase (PDI) and 1-Cys peroxiredoxin (1-Cys Prx) after transient focal cerebral ischemia in the rat brain [167]. Moreover, pretreatment of p-Hydroxybenzyl alcohol promoted functional recovery as indicated by the neurological severity score [166]. It also increased the expressions of PDI, nuclear factor-E2-related factor 2 (Nrf2), and several neurotrophic factor genes including glial cell line-derived neurotrophic factor (GDNF), brain-derived neurotrophic factor (BDNF), nerve growth factor (NGF), and myelin basic protein (MBP) genes [166]. The results indicate that p-Hydroxybenzyl alcohol can protect against neuroinflammation and brain damage.

## 7. Conclusions

The unsatisfactory outcome of existing drugs leads to the exploration of alternative medicines for treating acute and chronic neurodegenerative diseases. Traditional Chinese medicines used as alternative medicines are gaining more attention in western countries for curing various kinds of diseases. They are believed to be empirically effective and safe for thousands of years. With the help of modern experiment-based research, the compounds that are responsible for the biological effects of the herbs are isolated and their molecular modes of action are also characterized. In this review, we summarize the pharmacological effects of five groups of compounds including alkaloids, flavonoids, iridoids, carotenoids, and natural phenols from GUD. These compounds exert strong anti-inflammatory effect by inhibiting the production of cytokines, chemokines, and proteases in neuronal cells. In addition to the anti-inflammatory effect, they also exert strong antioxidative and antiapoptotic effects by reducing the generation of ROS, resulting in the reduction of necrosis and apoptosis of the neuronal cells.

## Figures and Tables

**Table 1a:** (a)

Alkaloids			
Rhynchophylline	*In vitro*/*ex vivo *		
Cells used (*in vitro*)	Inducer(s)	Functions	References

*Xenopus* oocytes	Glutamate	Inhibitory effects on NMDA receptors	[60]
Hippocampal slices *Xenopus* oocytes	Deprivation of oxygen and glucose	↓ neuronal damage Inhibitory effects on NMDA, muscarinic M_1_, and 5-HT_2_ receptors-mediated neurotoxicity	[62]
Rat cortical microglia	LPS	↓ nitric oxide production	[54]
Mouse N9 microglia	LPS	↓ TNF-*α*, IL1-*β*, and nitric oxide productions ↓ ERK and p38 phosphorylations, I*κ*B*α* degradation, and iNOS protein level	[55]
Rat primary microglia	LPS	↓ iNOS and COX-2 mRNA levels ↓ nitric oxide, PGE_2_, MCP-1, TNF-*α*, and IL1-*β* productions ↓ ERK and p38 phosphorylations and I*κ*B*α* degradation	[56]
NT2 cells	Dopamine	↓ apoptosis	[61]
Rat primary cortical neurons	Methamphetamine	↓ neurotoxicity	[63]
Rat cerebellar granule cells	Glutamate	↑ cell viability by inhibition of Ca^2+^ influx	[59]

Rhynchophylline	*In vivo *		
Type of animals (*in vivo*)	Disease model used (*in vivo*)	Functions	References

Rats	Kainic acid-induced epileptic seizures	↓ superoxide anions level, JNK phosphorylation, and NF-*κ*B activation	[57]

Isorhynchophylline	*In vitro*/*ex vivo *		
Cells used (*in vitro*)	Inducer(s)	Functions	References

*Xenopus* oocytes	Glutamate	Inhibitory effects on NMDA receptors by acting as noncompetitive antagonists	[60]
Hippocampal slices *Xenopus* oocytes	Deprivation of oxygen and glucose	↓ neuronal damage Inhibitory effects on NMDA, muscarinic M_1_, and 5-HT_2_ receptors-mediated neurotoxicity	[62]
N2a, SH-SY5Y, PC12 cells, and primary cortical neurons Differentiated human dopaminergic neurons	Nil	Stimulate autophagy of wild-type, A53T and A30P *α*-syn monomers, *α*-syn oligomers, and *α*-syn/synphilin-1 aggresomes ↓ wild-type and A53T *α*-syn protein expressions	[64]
Rat cortical microglia	LPS	↓ nitric oxide production	[54]
Mouse N9 microglia	LPS	↓ TNF-*α*, IL1-*β*, and nitric oxide productions ↓ ERK and p38 phosphorylations, I*κ*B*α* degradation, and iNOS protein level	[55]
PC12 cells	A*β*_(25–35)_	↑ cell viability and GSH level ↓ intracellular ROS and MDA levels ↓ DNA fragmentation and caspase-3 activity Stabilize mitochondrial membrane potential Moderate Bcl-2/Bax ratio	[58]
Rat cerebellar granule cells	Glutamate	↑ cell viability by inhibition of Ca^2+^ influx	[59]

Leonurine	*In vitro*/*ex vivo *		
Cells used (*in vitro*)	Inducer(s)	Functions	References

SH-SY5Y cells	6-OHDA	↓ cell death, ROS level, and Bax expression ↑ superoxide dismutase activity and Bcl-2 expression	[67]

Leonurine	*In vivo *		
Type of animals (*in vivo*)	Disease model used (*in vivo*)	Functions	References

Rats	MCAO	↓ ROS level and Bax expression ↑ Bcl-2 expression	[68]
Rats	MCAO	↓ infarct volume and lipid peroxidation ↑ superoxide dismutase and glutathione peroxidase	[69]

**Table 1b:** (b)

Flavonoids			
Catechin	*In vitro*/*ex vivo *		
Cells used (*in vitro*)	Inducer(s)	Functions	References

Rat cerebellar granule cells	Glutamate	↑ cell viability by inhibition of Ca^2+^ influx	[78]
Rat primary mesencephalic cultures	MPP^+^ H_2_O_2_, 4-HNE, rotenone, and 6-OHDA	↓ apoptosis ↑ cellular viability and [^3^H] DA uptake	[75]

Catechin	*In vivo *		
Type of animals (*in vivo*)	Disease model used (*in vivo*)	Functions	References

Rats	Nil	↓ MOA-B activity	[74]

Quercetin	*In vitro*/*ex vivo *		
Cells used (*in vitro*)	Inducer(s)	Functions	References

N9 microglia PC12 cells	LPS LPS-induced N9 microglia cells	↓ TNF-*α* and IL-1*α* mRNA levels ↓ apoptosis and cell death	[72]
PC12 cells	6-OHDA	↓ apoptosis and cell death ↓ nitric oxide overproduction and iNOS overexpression	[73]
Rat primary mesencephalic cultures	MPP^+^	↓ apoptosis	[75]
Rat cortical neuronal cultures	A*β*_(1–42)_	↓ cytotoxicity, protein oxidation, lipid peroxidation, and apoptosis	[79]
PC12 cells	MPP^+^	↓ apoptosis and cell death ↓ Bax and ↑ Bcl-2 expressions ↓ AIF in cytosolic and nuclear fraction ↓ cytochrome c levels in cytosolic fraction	[80]
P19 neurons	H_2_O_2_	↑ neuronal viability ↓ ROS production ↓ nuclear condensation, caspase 3/7 activity, and PARP upregulation	[76]
SH-SY5Y cells	H_2_O_2_	↓ cytotoxicity and LDH release ↓ Bax and ↑ Bcl-2 expressions ↓ caspase activation	[81]

Quercetin	*In vivo *		
Type of animals (*in vivo*)	Disease model used (*in vivo*)	Functions	References

Zebra fish	6-OHDA	↓ dopaminergic neuron loss ↓ TNF-*α*, IL-1*β*, and COX-2 overexpressions	[73]
Rats	6-OHDA	↑ striatal dopamine and antioxidant enzyme levels ↓ protein carbonyl content in the striatum ↑ neuronal survivability	[77]
Rats	Repeated cerebral ischemia	Improve spatial memory impairment ↓ neuronal death	[82]
Rats	pMCAO	↓ ischemic lesion ↑ GSH levels in ipsilateral striatum and cortex	[83]

Rutin	*In vitro/ex vivo *		
Cells used (*in vitro*)	Inducer(s)	Functions	References

RAW 264.7 cells	LPS	↓ nitric oxide production ↓ iNOS gene expression	[89]
SH-SY5Y cells BV-2 microglia	A**β**_42_	↓ ROS production ↓ TNF-*α* and IL-1*β* productions	[90]

Rutin	*In vivo *		
Type of animals (*in vivo*)	Disease model used (*in vivo*)	Functions	References

Rats	6-OHDA	↑ antioxidant enzymes activities ↓ nitric oxide level	[91]
Rats	Trimethyltin (TMT)	↓ IL-1*β* and IL-6 mRNA levels	[92]
Rats	Cerebral ischemia	↓ neuronal death	[93]

Baicalein	*In vitro/ex vivo *		
Cells used (*in vitro*)	Inducer(s)	Functions	References

BV-2 microglia	Hypoxia	↓ HIF-1 protein accumulation and transcriptional activation ↓ iNOS, COX-2, and VEGF gene expressions	[97]
Primary midbrain neuron-glia cultures	LPS	↓ TNF-*α*, nitric oxide, and superoxide productions	[94]
SH-SY5Y cells	6-OHDA	↓ oxidative stress, mitochondrial dysfunction, caspase activity, and JNK activation	[99]
SH-SY5Y cells	6-OHDA	↓ apoptosis	[100]
HT22 mouse hippocampal neuronal cells	Thapsigargin (TG) and brefeldin A (BFA)	↓ apoptosis ↓ C/EBP homologous protein (CHOP) induction and ROS accumulation	[101]
Rat glioma C6 cells	H_2_O_2_	↓ ROS-mediated cytotoxic effects Modulate ERKs activation ↑ HO-1 protein expression	[102]
Primary microglia/BV-2 cells	LPS/IFN-*γ*	↓ nitric oxide production and iNOS gene expression ↓ NF-IL6 binding	[98]
PC12 cells	Rotenone	↓ apoptosis ↓ ROS production	[103]

Baicalein	*In vivo *		
Type of animals (*in vivo*)	Disease model used (*in vivo*)	Functions	References

Rats	Controlled cortical impact injury	Improve functional recovery ↓ contusion volumes ↓ the number of degenerating neurons ↓ TNF-*α*, IL-1*β*, and IL-6 mRNA and protein levels	[104]

Baicalin	*In vitro/ex vivo *		
Cells used (*in vitro*)	Inducer(s)	Functions	References

RAW 264.7 cells	LPS	↓ nitric oxide production ↓ iNOS and COX-2 gene expressions	[118]
RAW 264.7 cells and peritoneal macrophages	LPS or IFN-*γ*	↓ nitric oxide production and iNOS expression ↓ TNF-*α*, ET-1, and thromboxane A2 (TXA2)	[106]
PC12 cells	Oxygen-glucose deprivation/H_2_O_2_	↓ ROS production ↓ 5-LOX nuclear translocation ↓ p38 phosphorylation	[107]

Baicalin	*In vivo *		
Type of animals (*in vivo*)	Disease model used (*in vivo*)	Functions	References

Rats	pMCAO	↓ neurological deficit scores and cerebral infarct volume ↓ iNOS, COX-2 mRNA, and cleaved caspase-3 protein expressions ↓ TLR2/4 and NF-*κ*B expressions	[108, 109]
Rats	Spinal cord injury	↓ oxidant stress, proinflammatory cytokines expressions, and apoptosis	[111]
Rats	Focal cerebral ischemic reperfusion injury	↓ NF-*κ*B p65 level	[110]

Wogonin	*In vitro/ex vivo *		
Cells used (*in vitro*)	Inducer(s)	Functions	References

RAW 264.7 cells	LPS	↓ PGE_2_ and nitric oxide productions ↓COX-2 expression and activity	[112–115]
Microglia	LPS	↓ nitric oxide production ↓ TNF-*α* and IL-6 productions ↓ NF-*κ*B activity	[116]
Microglia	MCP-1	↓ NF-*κ*B activity	[117]

Wogonin	*In vivo *		
Type of animals (*in vivo*)	Disease model used (*in vivo*)	Functions	References

Mice	LPS	↓ nitric oxide production ↓ iNOS expression	[113]
Rats	pMCAO	↓ infarct volume Improve behavioral dysfunction	[119]

Oroxylin A	*In vitro/ex vivo *		
Cells used (*in vitro*)	Inducer(s)	Functions	References

RAW 264.7 cells	LPS	↓ nitric oxide production ↓ iNOS and COX-2 gene expressions ↓ NF-*κ*B activation	[120]

Apigenin	*In vitro/ex vivo *		
Cells used (*in vitro*)	Inducer(s)	Functions	References

RAW 264.7 cells	LPS	↓ COX-2 and iNOS expressions ↓ NF-*κ*B activation	[123]
PBMC	LPS	↓ TNF-*α*, IL-6, and IL-1*β* productions	[124]
J774.2 macrophages	LPS	↓ TNF-*α* and IL-1*β* mRNA levels	[125]
BV-2 microglia	LPS	↓ nitric oxide and PGE_2_ productions ↓ p38 and JNK phosphorylations	[126]

Apigenin	*In vivo *		
Type of animals (*in vivo*)	Disease model used (*in vivo*)	Functions	References

Mice	MCAO	↓ infarct volume ↓ the number of microglia	[126]

Kaempferol	*In vitro/ex vivo *		
Cells used (*in vitro*)	Inducer(s)	Functions	References

J774.2 macrophages	LPS	↓ TNF-*α* and IL-1*β* mRNA levels	[125]
J774 macrophages	LPS	↓ PGE_2_ production ↓ COX-2 and mPGES-1 mRNA levels	[127]
J774 macrophages	LPS	↓ nitric oxide production ↓ iNOS mRNA and protein expressions	[128]
RAW 264.7 cells	LPS	↓ nitric oxide, PGE_2_, and TNF-*α* productions	[129]

Kaempferol	*In vivo *		
Type of animals (*in vivo*)	Disease model used (*in vivo*)	Functions	References

Rats	Transient focal cerebral ischemia	↓ nitrosative-oxidative stress, protein nitrotyrosines, and apoptotic cell death	[130]

Hyperoside	*In vitro/ex vivo *		
Cells used (*in vitro*)	Inducer(s)	Functions	References

PC12 cells	Sodium azide	↓ ROS production ↓ caspase-3 activity and Bax expression ↑ Bcl-2 expression	[133]
PC12 cells	H_2_O_2_ and tert-butyl hydroperoxide	↑ cell viability ↓ apoptosis	[132]
Mouse peritoneal macrophages	LPS	↓ TNF-*α*, IL-6, and nitric oxide productions	[131]

Hyperoside	*In vivo *		
Type of animals (*in vivo*)	Disease model used (*in vivo*)	Functions	References

Rats	MCAO	↓ infarct size and cerebral edema	[134]

**Table 1c:** (c)

Iridoids			
Geniposide	*In vitro/ex vivo *		
Cells used (*in vitro*)	Inducer(s)	Functions	References

PC12 cells	CoCl_2_	↓ apoptosis, Bax, P53, and caspase-9 expressions ↑ Bcl-2 expression	[147]
PC12 cells	SIN-1	↓ oxidative damage ↑ HO-1 expression	[139]
PC12 cells	H_2_O_2_	↑ Bcl-2 and HO-1 expressions	[140]
PC12 cells	H_2_O_2_	↓ oxidative damage ↑ Bcl-2 expression	[141]
Primary hippocampal neurons	SIN-1	↓ oxidative damage ↑ HO-1 expression	[142]
Rat hippocampal slice culture	Oxygen and glucose deprivation	↓ neuronal cell death	[148]

Genipin	*In vitro/ex vivo *		
Cells used (*in vitro*)	Inducer(s)	Functions	References

N2a cells	A23187	↓ cytotoxicity	[143]
N2a cells	6-OHDA	↓ neurotoxicity	[144]
Primary hippocampal neurons	A*β*_(25–35)_	↓ neurotoxicity	[145]
N2a cells	Tunicamycin	↑ cellular viability ↓ ER stress-induced upregulation of CHOP and GRP78	[146]
RAW 264.7 cells	LPS	↓ nitric oxide and PGE_2_ productions ↓ iNOS, COX-2, IL-6, IL-1*β*, and TNF-*α* expressions ↓ NF-*κ*B activation	[136]
Rat brain microglia	LPS IFN-*γ* and A*β*	↓ nitric oxide, TNF-*α*, IL-1*β*, PGE_2_, intracellular ROS productions, and NF-*κ*B activation ↓ nitric oxide release	[137]

Genipin	*In vivo *		
Type of animals (*in vivo*)	Disease model used (*in vivo*)	Functions	References

Mice and rats	Carrageenan	↓ paw edema, air pouch formation, and nitric oxide production	[138]
Mice	Carrageenan LPS	↓ paw edema ↓ plasma TNF-*α* and IL-6 productions	[136]
Mice	LPS	↓ microglial activation	[137]

**Table 1d:** (d)

Carotenoids			
Crocetin	*In vitro/ex vivo *		
Cells used (*in vitro*)	Inducer(s)	Functions	References

Isolated brain of stroke-prone spontaneously hypertensive rat	Nil	↓ ROS-mediated oxidative stress	[157]
SH-SY5Y cells	H_2_O_2_	↑ cellular viability ↓ ROS production and caspase-3 activation	[158]

Crocetin	*In vivo *		
Type of animals (*in vivo*)	Disease model used (*in vivo*)	Functions	References

Rats	6-OHDA	↑ antioxidant activity, GSH, and dopamine levels ↓ TBARS level	[159]

**Table 1e:** (e)

Natural phenols			
Gastrodin	*In vitro/ex vivo *		
Cells used (*in vitro*)	Inducer(s)	Functions	References

Cultured rat cortical neurons	Hypoxia	↑ neuron survival	[160]
Cultured rat hippocampal neurons	Oxygen/glucose deprivation and glutamate	↓ Ca^2+^ and nitric oxide productions	[161]
BV-2 cells	LPS	↓ TNF-*α* and IL-1*β* productions ↓ iNOS and COX-2 expressions	[162]

Gastrodin	*In vivo *		
Type of animals (*in vivo*)	Disease model used (*in vivo*)	Functions	References

Rats	MCAO	↓ cerebral infarct volume ↓ cerebral injury	[163]

*p*-Hydroxybenzyl alcohol	*In vitro/ex vivo *		
Cells used (*in vitro*)	Inducer(s)	Functions	References

RAW 264.7 cells	LPS	↓ nitric oxide production	[164]
BV-2 cells	LPS	↓ nitric oxide production	[165]
PC-12 cells	H_2_O_2_	↓ cell death	[166]

*p*-Hydroxybenzyl alcohol	*In vivo *		
Type of animals (*in vivo*)	Disease model used (*in vivo*)	Functions	References

Rats	MCAO	↓ brain damage ↑ protein disulfide isomerase (PDI) and 1-Cys peroxiredoxin (1-Cys Prx) transcription levels	[167]
Rats	MCAO	Modulate PDI and Nrf2 gene expressions and several neurotrophic factors	[166]
